# Complement Hyperactivation Is Mediated by Alternative and Lectin Pathways During Early Phase of Severe Vaccination‐Omicron BA.5 Infection

**DOI:** 10.1002/jmv.70863

**Published:** 2026-03-07

**Authors:** Jinpeng Cao, Gang Yang, Tingting Cui, Jian Qin, Deyi Huang, Shiqin Jin, Xiaoyun Yang, Mingzhu Huang, Xiaoling Su, Siyi Liu, Yingjiao Xia, Shidong Deng, Chengna Luo, Zhuxiang Zhao, Yunhui Zhang, Nanshan Zhong, Zhongfang Wang

**Affiliations:** ^1^ State Key Laboratory of Respiratory Disease & National Clinical Research Center for Respiratory Disease, Guangzhou Institute of Respiratory Health, the First Affiliated Hospital of Guangzhou Medical University Guangzhou Medical University Guangzhou Guangdong China; ^2^ Guangzhou National Laboratory Guangzhou International Bio Island Guangzhou Guangdong China; ^3^ Department of Pulmonary and Critical Care Medicine, The Affiliated Hospital of Kunming University of Science and Technology The First People's Hospital of Yunnan Province Kunming Yunnan China; ^4^ Department of Clinical Laboratory, State Key Laboratory of Respiratory Disease and National Clinical Research Centre for Respiratory Disease, The First Affiliated Hospital of Guangzhou Medical University Guangzhou Medical University Guangzhou Guangdong China; ^5^ South China University of Technology Guangzhou Guangdong China; ^6^ Department of Infectious Disease, Respiratory and Critical Care Medicine, Guangzhou First People's Hospital Guangzhou Medical University Guangzhou Guangdong China

**Keywords:** antibody response, complement immune response, COVID‐19 severity, thromboinflammation, vaccination

## Abstract

Complement temporal activation kinetics, activation pathways, and their relationship with thromboinflammation and antibody responses in severe vaccination‐COVID‐19 remains unclear. Based on a vaccinated‐infected cohort, we analyzed complement immune responses across mild to critical COVID‐19 cases by a dynamic model. Our results showed that complement activation was earlier (7–14 days post symptom onset), more intense and lasted longer in severe and critical vaccination‐COVID‐19, mediated by alternative and lectin pathways instead of classical pathway. Moreover, complement activation in severe vaccination‐COVID‐19 was related to thromboinflammation but not to the virus‐specific antibody response. Our study elucidated the kinetics of complement activation in vaccination‐COVID‐19 infection, revealing that Bb in alternative pathway were crucial in severe vaccination‐COVID‐19. These findings indicate the optimal timing for implementing complement activation‐targeted interventions in the management of severe vaccination‐COVID‐19 and provide more potential therapeutic targets.

## 1 Introduction

Acute respiratory infectious diseases significantly affect human health and economic development. Many emerging pathogens exploit the lungs as their primary site for viral replication and transmission, as demonstrated by the outbreaks of severe acute respiratory syndrome (SARS) in 2003, Middle East respiratory syndrome (MERS) in 2012, and the novel coronavirus disease (COVID‐19) in 2019, these highly pathogenic outbreaks often lead to profound pulmonary damage in patients [1]. In December 2022, the Omicron BA.5 variant was rapidly disseminated throughout southern China. The majority of vaccinated patients who were infected by Omicron BA.5 exhibited asymptomatic, mild, or moderate clinical manifestations, while a subset progressed to severe or critical cases. Complement activation, coagulative dysfunction, and inflammatory response in acute respiratory distress syndrome were closely associated with severe COVID‐19 [2, 3, 4, 5, 6, 7, 8, 9]. Excessive complement activation was closely associated with the increased mortality risk of COVID‐19 [4, 9, 10, 11]. Additionally, deposited complement factors, such as mannose‐binding protein‐associated serine protease‐2 (MASP‐2), complement Factor D (CFD), C3d, C1q, C4, and soluble membrane attack complex (sC5b‐9), had been observed in lung and kidney samples of patients with severe COVID‐19 [12, 13, 14]. However, it is unclear whether dynamic complement activation in the early‐ or late‐stage of COVID‐19 vaccine breakthrough infection influences disease severity differently; therefore, the timepoints of intervention to modulate complement activation remain unascertained.

Complement activation occurs via three major pathways: classical, lectin, and alternative. The classical pathway is initiated by C1q recognition of antigen‐antibody complexes, whereas the lectin pathway is triggered by mannose‐binding lectin (MBL) binding to pathogen‐associated carbohydrates; both pathways lead to C4 and C2 cleavage, forming the C3 convertase C4b2a. Cleavage of C4 generates C4d fragments, which serve as markers of classical and lectin pathway activation. In contrast, the alternative pathway is continuously primed by spontaneous hydrolysis of C3 to C3(H2O), a process known as “tick‐over.” C3(H2O) binds Factor B, which is cleaved by Factor D to form the initial fluid‐phase C3 convertase, C3(H2O)Bb. Deposition of C3b on target surfaces initiates an amplification loop through formation of surface‐bound C3 convertase (C3bBb), further stabilized by Factor B cleavage. Activation of all three pathways converges on C5 cleavage, producing the potent pro‐inflammatory peptides C3a and C5a and promoting assembly of the membrane attack complex. This ultimately leads to formation of the soluble membrane attack complex (sC5b‐9) through sequential incorporation of C5b, C6, C7, C8, and C9. In previous studies on complement activation pathways following SARS‐CoV‐2 infection, Castanha and Satyam et al. discovered that classical pathway plays a crucial role in complement activation [15, 16]; whereas Niederreiter et al. demonstrated that complement activation is mediated by the lectin pathway [12]; Niederreiter, Van Damme, Boussier, and Satyam et al. observed that alternative pathway is involved in complement activation [12, 16, 17, 18]. Despite the investigation of the complement activation pathway in several studies, the results still remained controversial [12, 15, 16, 17, 18]. All the aforementioned cross‐sectional studies primarily focused on unvaccinated individuals who were infected with SARS‐CoV‐2, wherein the primary antibody response was initiated later and of lower intensity compared to the recall antibody response in vaccinated people infected with the later‐emerging strains. As classical pathway of complement activation is antibody‐dependent, it will be interesting to explore whether the activation pathway differs in unvaccinated and vaccinated individuals infected with SARS‐CoV‐2.

The complement immune response, thrombosis, inflammatory response, and antibody response may be intricately interlinked in severe and critical vaccination‐COVID‐19, which offers a captivating research perspective for further exploration of the dynamic mechanisms underlying the complement immune response during early phase of severe COVID‐19 vaccine breakthrough infection. In this study, based on a vaccinated‐Omicron BA.5 infected cohort, we evaluated the levels of key complement factors (sC5b‐9, C3a, C5a, C1q, MBL, C4d, Bb, and Factor B) in the vaccinated patients with mild to critical COVID19 during acute infection. Moreover, we measured the dynamic changes of several thrombosis or inflammatory markers (e.g., ferritin, haptoglobin, and C‐reactive protein) to identify the upstream signal pathway that influences the complement‐activation terminal pathway and determine whether the complement immune response correlates with inflammation and thrombosis during acute infection. Lastly, we also investigated whether the levels of anti‐N + S/N IgG and Omicron BA.5 neutralizing antibodies neutralizing antibodies (Nabs) were involved in the classical pathway of complement activation.

## 2 Patients and Methods

### 2.1 Patient Cohort Characteristics

This study included a total of 125 patients with COVID‐19, confirmed by SARS‐CoV‐2 polymerase chain reaction (PCR) tests, who were recruited from the First People's Hospital of Yunnan Province and the First People's Hospital of Guangzhou between December 17, 2022, and May 1, 2023. Disease severity was assessed daily by reviewing electronic health records and classified according to the Diagnosis and Treatment Protocol for Novel Coronavirus Pneumonia (Trial Version 10) published by the National Health Commission of China. Mild disease status was characterized by the presence of only symptoms of upper respiratory tract infections, such as sore throat, cough, and fever. Moderate disease status was defined as: (1) hospitalization, (2) characteristic “COVID‐19 pneumonia” observed during imaging examination, and (3) a respiratory rate of < 30 times/min and oxygen saturation is > 93% when breathing air in the resting state. Severe disease status was characterized by the presence of the following features: (1) progressive exacerbation of clinical symptoms, and imaging examinations exhibiting a > 50% increase in pulmonary lesions within 24–48 h; (2) arterial partial pressure of oxygen (PaO_2_)/fraction of inspired oxygen (FiO_2_) ≤ 300 mmHg (1 mmHg = 0.133 kPa); and (3) respiratory rate ≥ 30 cycles/minor oxygen saturation ≤ 93%. Critical disease status was characterized by the presence of any of the following conditions: (1) respiratory failure necessitating mechanical ventilation, (2) shock, or (3) multiorgan failure necessitating ICU monitoring and treatment.

Basic patient information is presented in Supporting Information Table S1. Whole blood samples were collected, and un‐inactivated plasma samples were isolated from patients with mild COVID‐19 (*n* = 12) on days 14, 21, and 35 after symptom onset; and from patients with moderate (*n* = 45), severe (*n* = 40), and critical COVID‐19 (*n* = 28) on days 7, 14, 21, 28, and 35 post‐symptom onset, and each patient in acute phase provided 1–4 samples (Supporting Information Figure S1A). This study involved the analysis of inactivated plasma samples acquired from 54 unvaccinated and uninfected HDs (HC No Vaccination) between 2019 and 2020. For comparative purposes, the study incorporated un‐inactivated plasma samples collected from HDs who received three doses of CoronaVac (Sinovac Life Sciences Co., Ltd., Beijing, China) or BBIBP‐CorV (Sinopharm, Beijing, China) approximately 4 and 8 months earlier (I‐I‐I 4 Months, *N* = 19, and I‐I‐I 8 Months, *N* = 17). Comprehensive details of all healthy donors are presented in Supporting Information Table S2. This study received approval from the ethics committees of Yunnan Province's First People's Hospital (KHLL2023‐KY098) and Guangzhou First People's Hospital (NO.2021‐78). All the participants were provided with written informed consent.

### 2.2 Complement, Inflammatory, and Coagulative Factor Measurement

The levels of complement components, including sC5b‐9, C3a, C5a, C1q, MBL, C4d, Factor B, and Bb; coagulation and inflammatory factors, such as ferritin, haptoglobin, and C‐reactive protein, were measured using commercial ELISA kits (Jiangsu Meimian Industrial Co., Ltd, China) in cell‐free plasma samples from unvaccinated and uninfected HDs and patients with different COVID‐19 severities. The detection method was as follows: plasma samples were diluted 5 times with a sample diluent. The standard and sample wells were prepared. Standard substances of different concentrations (50 μL) were added to the standard wells, while the diluted test sample (50 μL) was added to the sample wells. Horseradish peroxidase (HRP)‐labeled detection antibody (100 μL) was added to each standard and sample well, followed by sealing with a plate film and incubation at 37°C for 60 min. The plate was washed 5 times with 350 μL washing solution. Substrates A and B (50 μL each) were then added to each well, followed by incubation at 37°C for 15 min in darkness. Finally, the termination solution (50 μL) was added to each well, and the absorbance at 450 nm was measured within 15 min using a Multiskan GO microplate spectrophotometer (Thermo Fisher Scientific, Waltham, MA, USA). The OD value of the standard was plotted on as the *x*‐axis, whereas the concentration value of the standard was used as the *y*‐axis to construct a standard curve and derive a binomial equation. The OD value of the sample was substituted into an equation to determine its concentration.

### 2.3 IFlash‐SARS‐CoV‐2 IgG Detection

The IFlash‐SARS‐CoV‐2 IgG test is a paramagnetic particle chemiluminescence immunoassay designed for the quantitative detection of Anti‐N + S SARS‐CoV‐2 IgG in plasma samples obtained from patients with different COVID‐19 severities, which was employed in a previous study [19]. This test was conducted according to the manufacturer's instructions (Shenzhen YHLO Biotechnology, Shenzhen, China). Cell‐free plasma was incubated with paramagnetic particles coated with SARS‐CoV‐2 antigen to ensure complete binding of the antibody to the coated antigen. Subsequently, acridine‐labeled anti‐human IgG conjugates were introduced to form reaction complexes. Following the addition of the trigger solution to the reaction mixture, the iFlash optical system was employed for determination of the antibody titer against Anti‐N + S SARS‐CoV‐2 IgG.

### 2.4 ELISA Detection of Anti‐N SARS‐CoV‐2 IgG

A direct ELISA can be utilized to detect anti‐N SARS‐CoV‐2 IgG in cell‐free inactivated plasma of patients with different COVID‐19 severities. A total of 100 ng SARS‐CoV‐2 N protein (Yiqiao Shenzhou, Cat#40588‐V08B‐1) was immobilized on ELISA plates (Jet, Cat#FEP‐200‐008) overnight at 4°C using a coating solution (Abcam, Cat#ab210899). Subsequently, the plates were blocked with PBS containing 5% skim milk and incubated at 37°C for 2 h. The standard sample comprised inactivated plasma with a high titer (500 units/mL) of anti‐N SARS‐CoV‐2 IgG. This standard sample was serially diluted two‐fold to evaluate other plasma samples, whereas the remaining plasma samples were diluted 10‐fold. The washing buffer served as the blank control. Each well of the ELISA plate received 100 μL of either the diluted standard sample or other plasma samples and was then incubated at 37°C for 40 min. An HRP‐labeled goat anti‐human IgG detection antibody (Yiqiao Shenzhou, Cat#SSA002), diluted to a concentration of 1:80 000 in PBS solution containing 2% skim milk, was added to each well and sealed with a plate cover before further incubation at 37°C for an additional 20 min. After discarding the liquid phase, each well underwent five washes using 350 μL washing solution. Next, 100 μL chromogenic substrate solution (Solarbio, Cat#PR1200) was added to each well and incubated at 37°C for 8–10 min under light‐shielded conditions. Finally, 50 μL termination solution (Solarbio, Cat#C1058‐500 mL) was introduced into each well prior to measuring absorbance at 450 nm within 15 min using a Multiskan GO microplate spectrophotometer (Thermo Fisher Scientific, Waltham, MA, USA). Data were analyzed using standard curves and logistic regression models.

### 2.5 SARS‐CoV‐2 Omicron BA.5 Virus Neutralization Test

Neutralization tests for SARS‐CoV‐2 Omicron BA.5 were conducted in a BSL‐3 certified laboratory, by following the methodology employed in previous studies [20, 21]. Initially, plasma samples were inactivated and tested at a dilution of 1:8. Subsequently, the samples were further diluted at eight points in 1:2 step increments. In each well of a 96‐well plate, 50 μL of the inactivated plasma sample was mixed with an equal volume of virus (100 TCID_50_). Following incubation at 37°C for 2 h, Vero E6 cells (1.2 × 10^4^, ATCC, USA) were seeded into these mixtures and cultured under humid conditions with 5% CO_2_ at 37°C for 4 days. Finally, the cytopathic effect (CPE) was assessed using a Celigo imaging cytometer by comparing each well with the corresponding positive and negative controls.

### 2.6 Statistics

Fitting and statistical analyses were conducted using GraphPad Prism software. Binomial nonlinear fitting analysis was used to analyze the parameters of complement immune response on days after symptom onset, with 95% confidence intervals. Binomial regression curves of immune parameters in patients with mild, moderate, severe, and critical COVID‐19 are shown in light gray, light yellow, light blue, and light red, respectively. Statistical significance was set at *p* < 0.05 (*p < 0.05; **p < 0.01; ***p < 0.001). The Mann–Whitney *U* test was employed to compare the central tendencies of the two groups (mean or median); all data are presented as median and interquartile ranges (IQR), and the geometric mean titers (GMT) were reported. Spearman correlation coefficients were employed to evaluate correlations.

## 3 Results

### 3.1 Earlier, Stronger, and Longer Complement Activation in Severe Vaccination‐COVID‐19

**Figure 1:**
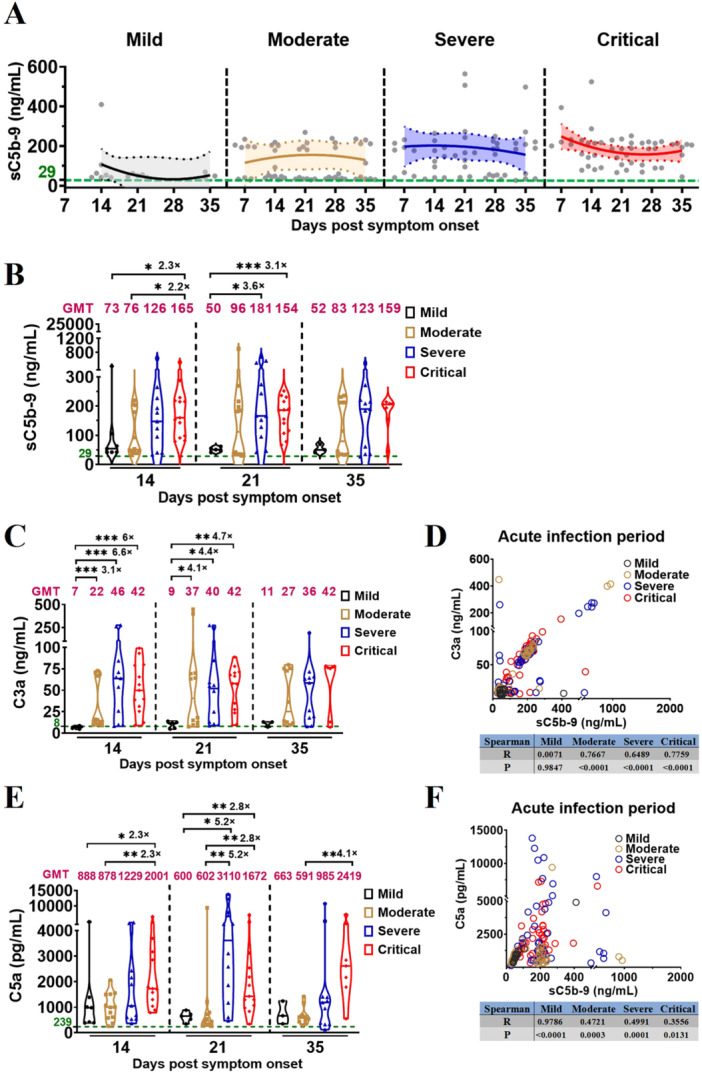
Earlier, stronger, and longer complement activation in severe vaccination‐COVID‐19. (A) Kinetics of sC5b‐9 levels in vaccinated patients with different COVID‐19 severities during acute infection. The 95% confidence intervals and binomial regression curve of sC5b‐9 levels in vaccinated patients with mild, moderate, severe, and critical COVID‐19 are shown in light gray, light yellow, light blue, and light red, respectively. (B) Comparison of sC5b‐9 levels in vaccinated patients with different COVID‐19 severities during acute infection. (C) Comparison of complement factor C3a levels in vaccinated patients with different COVID‐19 severities during acute infection. (D) Spearman correlation analysis of complement factor C3a and sC5b‐9 in vaccinated patients with different COVID‐19 severities during acute infection. (E) Comparison of complement factor C5a levels in vaccinated patients with different COVID‐19 severities during acute infection. (F) Spearman correlation analysis of complement factor C5a and sC5b‐9 in vaccinated patients with different COVID‐19 severities during acute infection. The magenta number indicates the geometric mean titers (GMT) of complement factor sC5b‐9/C3a/C5a in vaccinated patients with different COVID‐19 severities at the indicated timepoints, and fold‐change in GMTs for the sC5b‐9/C3a/C5a were compared with group with lower sC5b‐9/C3a/C5a titer. The number of vaccinated patients at the indicated timepoints: mild, 14, 21, 35 dps (*n* = 7, 5, 3); moderate, 7, 14, 21, 28, 35 dps (*n* = 8, 12, 12, 12, 10); severe, 7, 14, 21, 28, 35 dps (*n* = 9, 11, 12, 12, 11); critical, 7, 14, 21, 28, 35 dps (*n* = 5, 13, 13, 10, 7). Significance was measured using the Mann–Whitney *U* tests, *p < 0.05, **p < 0.01, ***p < 0.001. The green number and dotted line on the *y*‐axis represent the GMT of complement factor sC5b‐9 (29 ng/mL), C3a (8 ng/mL), and C5a (239 pg/mL) in 54 unvaccinated and uninfected healthy donors.

To investigate whether the complement immune response correlates to the disease severity of vaccination‐COVID‐19, we used ELISA to measure levels of the complement factor sC5b‐9 in plasma samples obtained from all healthy donors (HDs) and the vaccinated patients with different COVID‐19 severities (Supporting Information Tables S1 and S2). Based on sC5b‐9 kinetics during acute infection of Omicron BA.5, we showed a significant increase in plasma sC5b‐9 levels among vaccinated patients with mild to critical COVID‐19 compared to that in HDs (Figure 1A; Supporting Information Table S3). sC5b‐9 kinetics revealed that the complement activation was most pronounced in vaccinated patients with mild and moderate COVID‐19 at 14 and 21 days post symptom onset (D14 and D21), respectively. Conversely, in vaccinated patients with severe and critical COVID‐19, activation peaked at D14 and D7, respectively (Figure 1A; Supporting Information Table S3). Comparing the plasma sC5b‐9 levels at the same timepoints across different disease‐severity groups revealed an earlier, more intense, and longer activation of the complement immune response in those with severe and critical vaccination‐COVID‐19 (Figure 1B; Supporting Information Table S4), a significant difference between the critical and mild/moderate groups at D14 (165 vs. 73, 2.3‐fold, *p* = 0.0186; 165 vs. 76, 2.2‐fold, *p* = 0.0160), and between severe/critical and mild groups at D21 (181 vs. 50, 3.6‐fold, *p* = 0.0136; 154 vs. 50, 3.1‐fold, *p* = 0.0002). In addition, compared with unvaccinated and uninfected HDs, plasma sC5b‐9 levels did not increase in uninfected HDs who received three doses of inactivated vaccines at the indicated timepoints (Supporting Information Figure S1B), thus the administration of three doses of inactivated vaccine does not activate complement immune responses in HDs.

To further investigate the conversion rates of C3 and C5, which are upstream of sC5b‐9 in the complement‐activation signaling pathway, C3a and C5a levels were measured using ELISA in plasma samples obtained from patients with different COVID‐19 severity and HDs. Our results revealed a significant increase in the plasma levels of C3a and C5a in vaccinated patients with severe and critical disease than that of vaccinated patients with mild/moderate, and the HDs (Figure 1C,E; Supporting Information Table S4). Comparison of the plasma levels of C3a and C5a at the same timepoints across different disease‐severity groups (Figure 1C,E; Supporting Information Table S4) showed a significant difference in the C3 conversion rate between the moderate/severe/critical and mild groups on D14 (22 vs. 7, 3.1‐fold, *p* < 0.0001; 46 vs. 7, 6.6‐fold, *p* = 0.0001; 42 vs. 7, 6.0‐fold, *p* < 0.0001) and D21 (37 vs. 9, 4.1‐fold, *p* = 0.0485; 40 vs. 9, 4.4‐fold, *p* = 0.0365; and 42 vs. 9, 4.7‐fold, *p* = 0.0068). Moreover, the C5 conversion rate significantly differed between the critical and mild/moderate COVID‐19 groups on D14 (2001 vs. 888, 2.3‐fold, *p* = 0.0456; 2001 vs. 878, 2.3‐fold, *p* = 0.0055); severe/critical and mild groups on D21 (3110 vs. 600, 5.2‐fold, *p* = 0.0136; 1672 vs. 600, 2.8‐fold, *p* = 0.0068); severe/critical and moderate groups on D21 (3110 vs. 602, 5.2‐fold, *p* = 0.0014; 1672 vs. 602, 2.8‐fold, *p* = 0.0024); and critical and moderate groups on D35 (2419 vs. 591, 4.1‐fold, *p* = 0.0020). Correlation analysis demonstrated a strong association between complement activation and the C3 conversion rate in vaccinated patients with moderate, severe, and critical COVID‐19 during acute‐phase infection (Figure 1D). Complement activation during the acute infection highly correlated with C5 conversion rates in vaccinated patients with different COVID‐19 severities (Figure 1F).

### 3.2 Complement Activation in Severe Vaccination‐COVID‐19 Is Mediated by Alternative Pathway

Both generations of Bb and cleavage of Factor B play an important role in alternative pathway. To investigate which upstream signal pathway affects the terminal pathway for complement activation in different severities of vaccination‐COVID‐19 and HDs, we evaluated the levels of Bb and Factor B in plasma samples from vaccinated patients with different COVID‐19 severities and HDs using ELISA.

**Figure 2:**
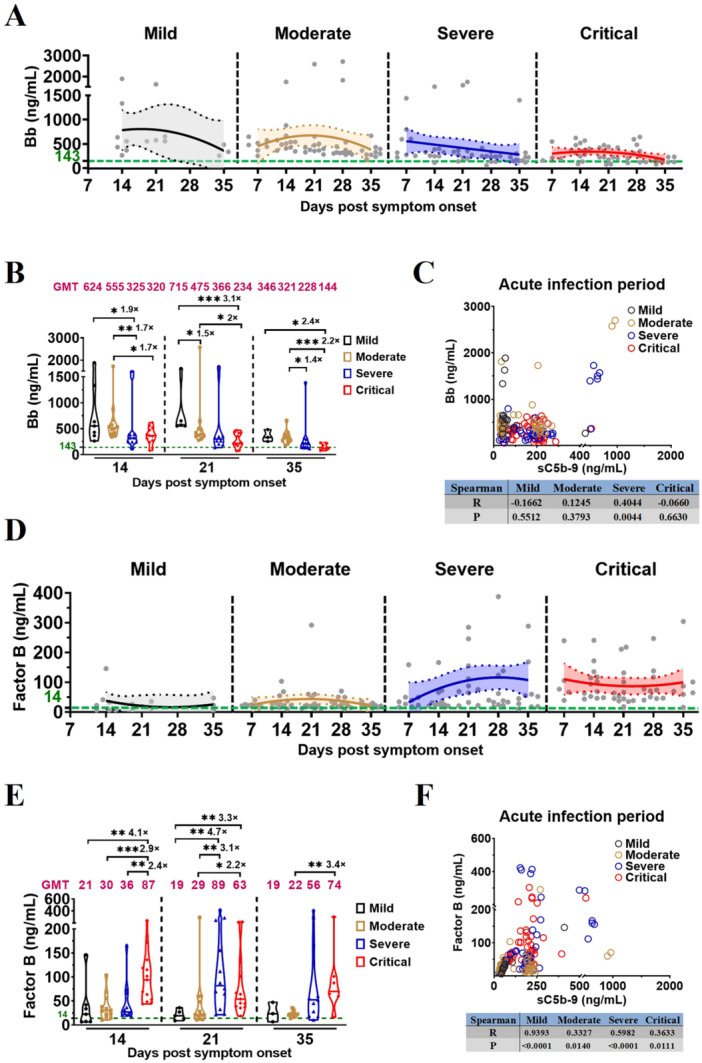
Complement activation in severe vaccination‐COVID‐19 is mediated by alternative pathway. (A) Kinetics of Bb levels in vaccinated patients with different COVID‐19 severities during acute infection. The 95% confidence intervals and binomial regression curve of Bb levels in vaccinated patients with mild, moderate, severe, and critical COVID‐19 are shown in light gray, light yellow, light blue, and light red, respectively. (B) Comparison of Bb levels in vaccinated patients with different COVID‐19 severities during acute infection. (C) Spearman correlation analysis of complement Bb and sC5b‐9 in vaccinated patients with different COVID‐19 severities during acute infection. (D) Kinetics of Factor B levels in vaccinated patients with different COVID‐19 severities during acute infection. The 95% confidence intervals and binomial regression curve of Factor B levels in vaccinated patients with mild, moderate, severe, and critical COVID‐19 are shown in light gray, light yellow, light blue, and light red, respectively. (E) Comparison of Factor B levels in vaccinated patients with different COVID‐19 severities during acute infection. (F) Spearman correlation analysis of complement Factor B and sC5b‐9 in vaccinated patients with different COVID‐19 severities during acute infection. The magenta number indicates the geometric mean titers (GMT) of complement Bb/Factor B in vaccinated patients with different COVID‐19 severities at the indicated timepoints, and fold‐change in GMTs for the Bb/Factor B was compared with the group with lower Bb/Factor B titer. The number of vaccinated patients at the indicated timepoints: mild, 14, 21, 35 dps (*n* = 7, 5, 3); moderate, 7, 14, 21, 28, 35 dps (*n* = 8, 12, 12, 12, 10); severe, 7, 14, 21, 28, 35 dps (*n* = 9, 11, 12, 12, 11); critical, 7, 14, 21, 28, 35 dps (*n* = 5, 13, 13, 10, 7). Significance was measured using the Mann–Whitney *U* tests, *p < 0.05, **p < 0.01, ***p < 0.001. The green number and dotted line on the *y*‐axis represent the GMT of complement Bb (143 ng/mL) and Factor B (14 ng/mL) in 54 unvaccinated and uninfected healthy donors.

Bb and Factor B kinetics demonstrated a significant elevation in the plasma levels of Bb and Factor B among vaccinated patients with different severities of COVID‐19 when compared to HDs, with Factor B consumption being tightly linked to Bb formation (Figure 2A,D; Supporting Information: Figure S1C and Table S3). Comparison of plasma Bb levels at the same time points across disease‐severity groups revealed significantly higher Bb levels in vaccinated patients with mild/moderate COVID‐19 (Figure 2B; Supporting Information Table S4); plasma levels of Bb significantly differed between vaccinated patients with: mild/moderate and severe COVID‐19 on D14 (624 vs. 325, 1.9‐fold, *p* = 0.04; 555 vs. 325, 1.7‐fold, *p* = 0.0042); moderate and critical COVID‐19 on D14 (555 vs. 320, 1.7‐fold, *p* = 0.016); mild/moderate and critical COVID‐19 on D21 (715 vs. 234, 3.1‐fold, *p* = 0.0002; 475 vs. 234, 2‐fold, *p* = 0.0258); mild and moderate COVID‐19 on D21 (715 vs. 475, 1.5‐fold, *p* = 0.0275); mild/moderate and critical COVID‐19 on D35 (346 vs. 144, 2.4‐fold, *p* = 0.0167; 321 vs. 144, 2.2‐fold, *p* = 0.0007); moderate and severe COVID‐19 on D35 (321 vs. 228, 1.4‐fold, *p* = 0.0242); Correlation analysis further demonstrated a strong association of the complement activation with the Bb during acute infection in severe vaccination‐COVID‐19 (Figure 2C).

Moreover, comparison of plasma Factor B levels at the same time points across disease‐severity groups revealed significantly higher Factor B levels in vaccinated patients with severe and critical COVID‐19 (Figure 2E; Supporting Information Table S4); plasma levels of Factor B significantly differed between vaccinated patients with: critical and mild/moderate/severe COVID‐19 on D14 (87 vs. 21, 4.1‐fold, *p* = 0.0047; 87 vs. 30, 2.9‐fold, *p* = 0.0005; 87 vs. 36, 2.4‐fold, *p* = 0.0031); severe/critical and mild COVID‐19 on D21 (89 vs. 19, 4.7‐fold, *p* = 0.0061; 63 vs. 19, 3.3‐fold, *p* = 0.0028); severe/critical and moderate COVID‐19 on D21 (89 vs. 29, 3.1‐fold, *p* = 0.0029; 63 vs. 29, 2.2‐fold, *p* = 0.0298); and critical and moderate COVID‐19 on D35 (74 vs. 22, 3.4‐fold, *p* = 0.0097). Correlation analysis further demonstrated a strong association of the complement activation with the Factor B during acute infection in vaccinated patients with different COVID‐19 severities (Figure 2F).

### 3.3 Complement Activation in Severe Vaccination‐COVID‐19 Is Mediated by Lectin Pathway Instead of Classical Pathway

Both generations of C4d and consumption of MBL/C1q play a crucial role in the lectin or classical pathway in complement activation. To investigate whether the lectin or classical pathway was involved in the complement activation, thereby influencing vaccination‐COVID‐19 severity, the levels of complement factor C4d, MBL and C1q were ascertained using ELISA in plasma samples obtained from vaccinated patients with different COVID‐19 severity and HDs.

**Figure 3:**
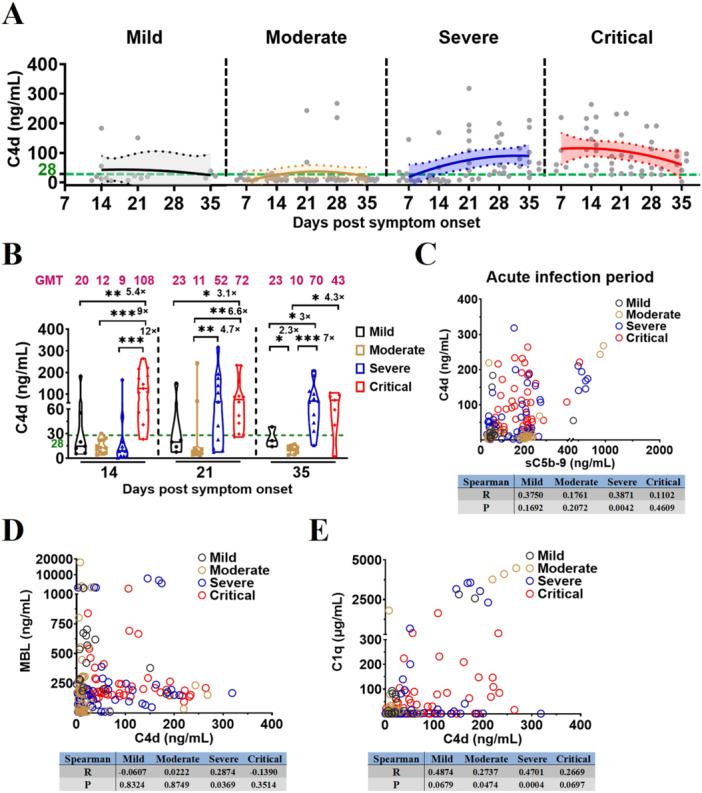
Complement activation in severe vaccination‐COVID‐19 is mediated by lectin pathway or classical pathway. (A) Kinetics of C4d levels in vaccinated patients with different COVID‐19 severities during acute infection. The 95% confidence intervals and binomial regression curve of C4d levels in vaccinated patients with mild, moderate, severe, and critical COVID‐19 were shown in light gray, light yellow, light blue, and light red, respectively. (B) Comparison of C4d levels in vaccinated patients with different COVID‐19 severities during acute infection. (C–E) Spearman correlation analysis of complement C4d and sC5b‐9/MBL/C1q in vaccinated patients with different COVID‐19 severities during acute infection. The magenta number indicates the geometric mean titers (GMT) of complement factor C4d in vaccinated patients with different COVID‐19 severities at the indicated timepoints. The number of vaccinated patients at the indicated timepoints: mild, 14, 21, 35 dps (*n* = 7, 5, 3); moderate, 7, 14, 21, 28, 35 dps (*n* = 8, 12, 12, 12, 10); severe, 7, 14, 21, 28, 35 dps (*n* = 9, 11, 12, 12, 11); critical, 7, 14, 21, 28, 35 dps (*n* = 5, 13, 13, 10, 7). Significance was measured using the Mann–Whitney *U* tests. The green number and dotted line on the *y*‐axis represent the GMT of complement factor C4d (28 ng/mL) in 54 unvaccinated and uninfected healthy donors.

C4d kinetics demonstrated a significant elevation in the plasma levels of C4d among vaccinated patients with severe/critical COVID‐19 when compared to HDs (Figure 3A; Supporting Information Table S3). Comparison of plasma C4d levels at the same time points across disease‐severity groups revealed significantly higher C4d levels in vaccinated patients with severe/critical COVID‐19 (Figure 3B; Supporting Information Table S4); plasma levels of C4d significantly differed between vaccinated patients with: mild/moderate/severe and critical COVID‐19 on D14 (20 vs. 108, 5.4‐fold, *p* = 0.0047; 12 vs. 108, 9‐fold, *p* < 0.0001; 9 vs. 108, 12‐fold, *p* = 0.0001); mild/moderate and critical COVID‐19 on D21 (23 vs. 72, 3.1‐fold, *p* = 0.0268; 11 vs. 72, 6.6‐fold, *p* = 0.0011); moderate and severe COVID‐19 on D21 (11 vs. 52, 4.7‐fold, *p* = 0.0086); mild and moderate COVID‐19 on D35 (23 vs. 10, 2.3‐fold, *p* = 0.0490); moderate and critical COVID‐19 on D35 (10 vs. 43, 4.3‐fold, *p* = 0.0136); mild/moderate and severe COVID‐19 on D35 (23 vs. 70, 3‐fold, *p* = 0.0385; 10 vs. 70, 7‐fold, *p* < 0.0001); Correlation analyses further demonstrated that C4d levels were associated with the complement activation and MBL in severe vaccination‐COVID‐19, and were also associated with C1q in moderate and severe vaccination‐COVID‐19 during the acute phase of infection (Figure 3C–E).

**Figure 4:**
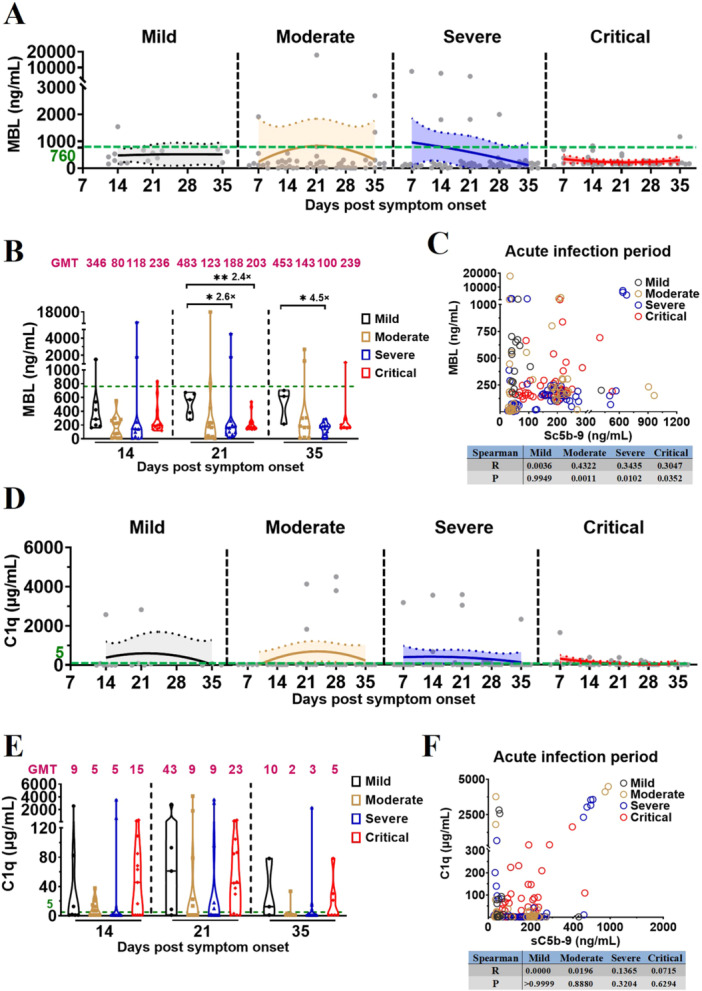
Complement activation in severe vaccination‐COVID‐19 is mediated by lectin pathway instead of classical pathway. (A) Kinetics of MBL levels in vaccinated patients with different COVID‐19 severities during acute infection. The 95% confidence intervals and binomial regression curve of MBL levels in vaccinated patients with mild, moderate, severe, and critical COVID‐19 were shown in light gray, light yellow, light blue, and light red, respectively. (B) Comparison of MBL levels in vaccinated patients with different COVID‐19 severities during acute infection. (C) Spearman correlation analysis of complement MBL and sC5b‐9 in vaccinated patients with different COVID‐19 severities during acute infection. (D) Kinetics of C1q levels in vaccinated patients with different COVID‐19 severities during acute infection. The 95% confidence intervals and binomial regression curve of C1q levels in vaccinated patients with mild, moderate, severe, and critical COVID‐19 are shown in light gray, light yellow, light blue, and light red, respectively. (E) Comparison of C1q levels in vaccinated patients with different COVID‐19 severities during acute infection. (F) Spearman correlation analysis of complement factor C1q and sC5b‐9 in vaccinated patients with different COVID‐19 severities during acute infection. The magenta number indicates the geometric mean titers (GMT) of complement factor MBL/C1q in vaccinated patients with different COVID‐19 severities at the indicated timepoints. The number of vaccinated patients at the indicated timepoints: mild, 14, 21, 35 dps (*n* = 7, 5, 3); moderate, 7, 14, 21, 28, 35 dps (*n* = 8, 12, 12, 12, 10); severe, 7, 14, 21, 28, 35 dps (*n* = 9, 11, 12, 12, 11); critical, 7, 14, 21, 28, 35 dps (*n* = 5, 13, 13, 10, 7). Significance was measured using the Mann–Whitney *U* tests. The green number and dotted line on the *y*‐axis represent the GMT of complement factor MBL (760 ng/mL) and C1q (5 μg/mL) in 54 unvaccinated and uninfected healthy donors.

Moreover, the MBL kinetic models revealed that, compared with HDs, plasma MBL levels decreased during acute infection in vaccinated patients with different COVID‐19 severities (Figure 4A; Supporting Information Table S3); Comparison of plasma MBL levels at the same time points across disease‐severity groups revealed significantly lower MBL levels in vaccinated patients with severe and critical COVID‐19 (Figure 4B; Supporting Information Table S4); plasma levels of MBL significantly differed between vaccinated patients with: mild and severe/critical COVID‐19 on D21 (483 vs. 188, 2.6‐fold, *p* = 0.0365; 483 vs. 203, 2.4‐fold, *p* = 0.0028); mild and severe COVID‐19 on D35 (453 vs. 100, 4.5‐fold, *p* = 0.0192). Correlation analyses further demonstrated that MBL levels were associated with the complement activation in moderate, severe and critical vaccination‐COVID‐19 during the acute phase of infection (Figure 4C).

**Figure 5:**
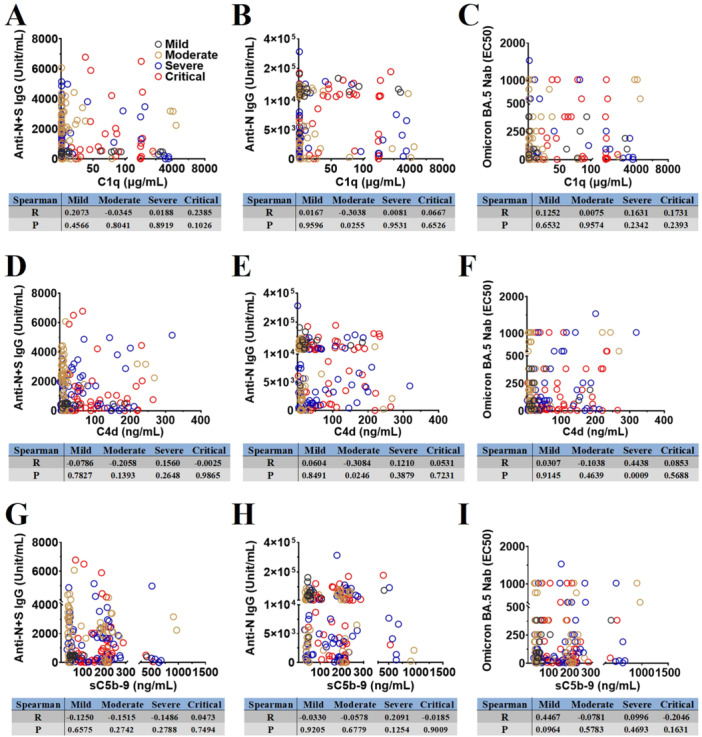
Spearman correlation analysis of complement factors C1q, C4d, sC5b‐9 with COVID‐19‐specific antibody response (Anti N + S/N IgG and Omicron BA.5 neutralizing antibodies) in vaccinated patients with different COVID‐19 severities during acute infection (A–I).

In addition, C1q kinetic models revealed that, during acute infection, C1q levels increased in vaccinated patients with different COVID‐19 severities compared with those in HDs (Figure 4D; Supporting Information Table S3); however the levels did not differ significantly between the vaccinated patients with severe/critical and mild/moderate COVID‐19 (Figure 4E; Supporting Information Table S4). Correlation analysis demonstrated that the C1q is not associated with the complement activation in the vaccinated patients with different COVID‐19 severities during acute infection (Figure 4F). To further confirm the relationship among complement activation, the classical pathway and the COVID‐19‐specific antibody response in vaccinated patients with different COVID‐19 severities, we assessed the plasma levels of Anti N + S IgG, Anti N IgG, and Omicron BA.5 Nabs in the vaccinated patients with different COVID‐19 severities using either ELISA or live virus in vitro neutralization assays, which were correlated with complement factors C1q, C4d, and sC5b‐9 in the classical and terminal pathways. Our results showed that C1q, C4d, and sC5b‐9 in vaccinated patients with different COVID‐19 severities were generally not correlated with the COVID‐19‐specific antibody response, such as anti‐N + S/N IgG and Omicron BA.5 Nabs, during acute infection (Figure 5A–I).

Overall, compared with HDs, the vaccinated patients with severe COVID‐19 exhibited pronounced plasma MBL consumption, which is strongly associated with the generation of C4d and the terminal complement complex sC5b‐9, supporting predominant complement activation through the lectin pathway in severe vaccination‐COVID‐19. In contrast, although C4d generation is mechanistically linked to C1q in the classical pathway, plasma C1q levels are increased and show no association with sC5b‐9 formation. Furthermore, levels of COVID‐19‐specific antibodies are generally not correlated with C1q, C4d, or sC5b‐9 in the vaccinated patients with different COVID‐19 severities. Notably, only in severe vaccination‐COVID‐19 is Nabs abundance associated with C4d generation, without a corresponding increase in terminal complement activation. Collectively, these results indicate that complement activation in severe vaccination‐COVID‐19 is mediated by lectin pathway instead of classical pathway.

### 3.4 Complement Activation in Severe Vaccination‐COVID‐19 Correlates to Thrombosis and Inflammatory Response

**Figure 6:**
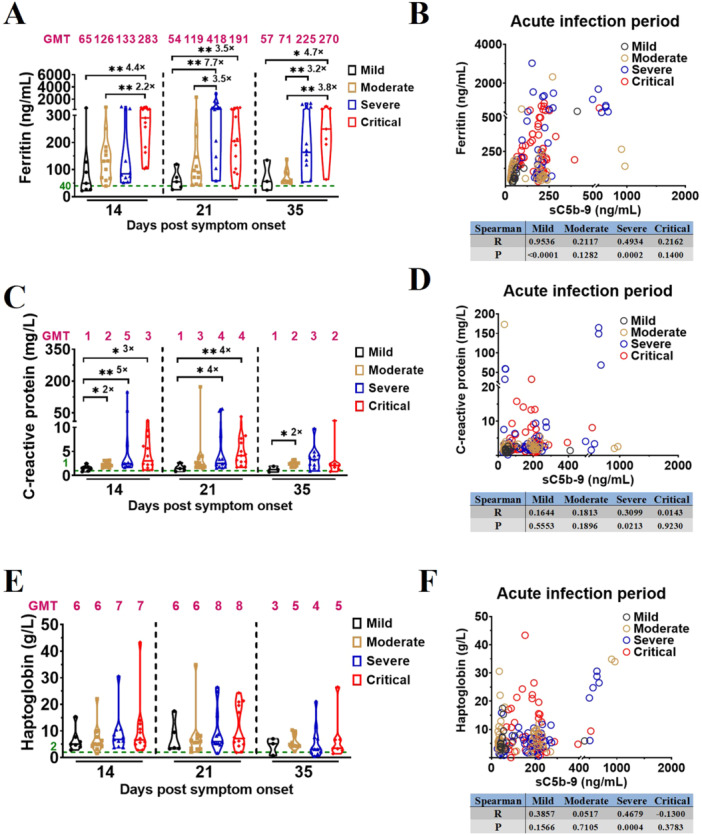
Complement activation in severe vaccination‐COVID‐19 correlates to thrombosis and inflammatory response. (A) Comparison of ferritin levels in vaccinated patients with different COVID‐19 severities during acute infection. (B) Spearman correlation analysis of ferritin and sC5b‐9 in vaccinated patients with different COVID‐19 severities during acute infection. (C) Comparison of C‐reactive protein levels in vaccinated patients with different COVID‐19 severities during acute infection. (D) Spearman correlation analysis of C‐reactive protein and sC5b‐9 in vaccinated patients with different COVID‐19 severities during acute infection. (E) Comparison of haptoglobin levels in vaccinated patients with different COVID‐19 severities during acute infection. (F) Spearman correlation analysis of haptoglobin and sC5b‐9 in vaccinated patients with different COVID‐19 severities during acute infection. The magenta number indicates the geometric mean titers (GMT) of ferritin/C‐reactive protein/haptoglobin in vaccinated patients with different COVID‐19 severities at the indicated timepoints, and fold‐change in GMTs for ferritin/C‐reactive protein was compared with the group with lower ferritin/C‐reactive protein titer. The number of vaccinated patients at the indicated timepoints: mild, 14, 21, 35 dps (*n* = 7, 5, 3); moderate, 7, 14, 21, 28, 35 dps (*n* = 8, 12, 12, 12, 10); severe, 7, 14, 21, 28, 35 dps (*n* = 9, 11, 12, 12, 11); critical, 7, 14, 21, 28, 35 dps (*n* = 5, 13, 13, 10, 7). Significance was measured using the Mann–Whitney *U* tests, *p < 0.05, **p < 0.01, ***p < 0.001. The green number and dotted line on the *y*‐axis represent the GMTs of ferritin (40 ng/mL), C‐reactive protein (1 mg/L), and haptoglobin (2 g/L) in 54 unvaccinated and uninfected healthy donors.

In order to elucidate the correlation between complement activation, thrombosis, and inflammatory response in the vaccinated patients with different COVID‐19 severities during early phase of SARS‐CoV‐2 infection, plasma ferritin, haptoglobin, and C‐reactive protein levels were assessed via ELISA in vaccinated patients with different COVID‐19 severities and HDs. During early phase of SARS‐CoV‐2 infection, vaccinated patients with different COVID‐19 severities exhibited elevated plasma levels of ferritin, haptoglobin, and C‐reactive protein than those in HDs (Figure 6A,C,E; Supporting Information Table S4).

Plasma levels of ferritin at the same timepoints across different disease‐severity groups (Figure 6A,C; Supporting Information Table S4) differed significantly between vaccinated patients with: critical and mild/moderate COVID‐19 on D14 (283 vs. 65, 4.4‐fold, *p* = 0.0063; 283 vs. 126, 2.2‐fold, *p* = 0.0066); severe/critical and mild COVID‐19 on D21 (418 vs. 54, 7.7‐fold, *p* = 0.0032; 191 vs. 54, 3.5‐fold, *p* = 0.0098); severe and moderate COVID‐19 on D21 (418 vs. 119, 3.5‐fold, *p* = 0.0129); critical and mild COVID‐19 on D35 (270 vs. 57, 4.7‐fold, *p* = 0.0333); and severe/critical and moderate COVID‐19 on D35 (225 vs. 71, 3.2‐fold, *p* = 0.0039; 270 vs. 71, 3.8‐fold, *p* = 0.0012). Furthermore, plasma levels of C‐reactive protein differed significantly between the moderate/severe/critical and mild COVID‐19 groups on D14 (2 vs. 1, 2‐fold, *p* = 0.0128; 5 vs. 1, 5‐fold, *p* = 0.0054; 3 vs. 1, 3‐fold, *p* = 0.0176), severe/critical and mild COVID‐19 groups on D21 (4 vs. 1, 4‐fold, *p* = 0.0268; 4 vs. 1, 4‐fold, *p* = 0.0044), and moderate and mild COVID‐19 groups on D35 (2 vs. 1, 2‐fold, *p* = 0.0245). Correlation analysis demonstrated a significant association of complement activation during acute infection with ferritin levels in vaccinated patients with mild and severe COVID‐19 (Figure 6B). Moreover, a strong correlation was observed between complement activation and C‐reactive protein or haptoglobin levels in vaccinated patients with severe COVID‐19 during acute infection (Figure 6D,F). A strong correlation was also observed between MBL/C1q and C‐reactive protein in vaccinated patients with severe or critical COVID‐19 (Supporting Information Figure S1D,E).

## 4 Discussion

Dysregulated complement activation has been identified as a key factor in the development of acute lung diseases induced by highly pathogenic viruses, including influenza A viruses (H1N1, H5N1, and H7N9) [22, 23, 24], severe acute respiratory syndrome coronavirus (SARS‐CoV) [25], and Middle East respiratory syndrome coronavirus (MERS‐CoV) [26]. Moreover, the inhibition of complement activation in mouse models of H1N1 or H5N1 infection induced suppression of airway neutrophil and macrophage infiltration, neutrophil extracellular traps (NETs), lung epithelial injury, and overall lung injury, ultimately improved survival rates in mice [22, 23]. Similarly, inhibition of complement activation was found to not only alleviate lung injury but also reduce inflammation and viral load in lung tissues, in both H7N9‐infected monkey models and MERS‐CoV‐infected mouse models [24, 27]. Additionally, in a murine model of SARS‐CoV infection, the absence of the *C3* gene decreased pulmonary neutrophilic infiltration and attenuated systemic inflammation [28]. Since the onset of COVID‐19 pandemic, a plethora of studies have consistently demonstrated a correlation between complement dysregulation and COVID‐19 severity [3, 29, 30]. The COVID‐19 severity directly affects the magnitude of complement activation [9, 18, 31]. However, these studies on complement primarily commenced during the early stages of the COVID‐19 epidemic, thereby failing to fully consider the impact of vaccine‐induced immunity and viral mutations on the complement immune response. Furthermore, previous studies [7, 12, 32, 33, 34, 35, 36] did not elucidate the kinetics of the complement immune response among vaccinated patients with varying degrees of COVID‐19 severity during acute infection. The optimal timing for complement intervention in severe vaccination‐COVID‐19 to prevent immune damage secondary to excessive complement activation remains an unresolved issue within this field.

Here, we have not only found that inactivated vaccine immunization had no effect on the complement immune response in healthy individuals, but also established a kinetic model of the complement immune response across different severities of vaccination‐COVID‐19 during acute infection, based on an inactivated vaccine immunization‐Omicron BA.5 infection cohort. We demonstrated that complement activation was broadly and robustly triggered during the acute phase across all levels of clinical severity in a retrospective cohort of individuals immunized with inactivated SARS‐CoV‐2 vaccines who subsequently experienced Omicron BA.5 breakthrough infection. Importantly, both the magnitude and the persistence of complement activation exhibited a clear positive correlation with disease severity, with the most pronounced responses observed in severe vaccination‐COVID‐19, highlighting a severity‐dependent complement response in the context of breakthrough infection under inactivated vaccine‐induced immunity. Furthermore, compared with those with mild/moderate vaccination‐COVID‐19, vaccinated patients with severe COVID‐19 had higher C3 or C5 conversion rates during acute infection, which were significantly associated with complement activation. These results aligned with those of previous studies and revealed a direct correlation between the severity of COVID‐19 and the intensity of complement activation [9, 17, 18, 31, 37]. Meanwhile, neither inactivated vaccine immunity nor SARS‐CoV‐2 mutation influenced the intensity of complement activation. Moreover, based on previous studies, we further observed that complement activation in vaccination‐COVID‐19 tended to occur earlier as the disease severity increased. Compared with vaccinated patients with mild and moderate COVID‐19, those with severe COVID‐19 exhibited the most robust complement activation between days 7 and 14 after acute infection. This elucidates the temporal mechanism of dysregulated complement immune response in vaccinated patients with severe COVID‐19, offering an optimal timing for clinical intervention for targeting the complement immune response in severe vaccination‐COVID‐19, thereby bridging a crucial gap within this field.

The classical, lectin, and alternative pathways all play pivotal roles in the signaling cascades triggered by complement activation. Our study revealed that vaccinated patients with different COVID‐19 severities activated their complement immune responses through alternative and lectin pathways during acute infection. In contrast, some early studies showed that the classical, lectin, and alternative pathways were all involved in COVID‐19 complement activation [12, 16, 38], which differs from the findings of our study. Activation of the classical pathway relies on a virus‐specific antibody response, and early studies mostly used a cohort consisting of unvaccinated individuals with primary COVID‐19 during the early pandemic phase. In contrast, our study was conducted in a vaccinated‐infected cohort, and participants who had received one or three doses of inactivated COVID‐19 vaccines exhibited a quicker increase and a higher level of COVID‐19‐specific antibodies following an Omicron BA.5 variant breakthrough infection. Theoretically, the antibody‐dependent classical pathway should be activated in the vaccinated cohort rather than in the primary infection cohort; however, our results did not show a correlation between the virus‐specific antibody response and complement activation. In fact, identifying the specific pathway involved in complement activation during vaccination‐viral breakthrough infections is complex and seldom studied. Prior to the COVID‐19 pandemic phase, only a study of H1N1 infection in mouse models demonstrated that Ficolin A in the lectin pathway can induce excessive complement activation and thereby exacerbates the H1N1 infection‐induced acute lung immunopathological injury [39]. Therefore, the underlying mechanism of complement activation‐dependent pathways in different contexts, including different viruses and disease outcomes, with and without pre‐existing immunity, should be further investigated in the future. In addition to traditional intervention targets, C3 or C5 inhibitors were used in the early stages of the COVID‐19 epidemic [40, 41, 42]. Herein, we proposed that Bb, one key components of the alternative pathway, can serve as important target for early intervention to mitigate excessive complement activation in severe vaccination‐COVID‐19.

We also investigated the association among complement activation, thrombosis, and inflammation, revealing that vaccinated patients with severe COVID‐19 exhibited heightened thrombotic and inflammatory responses during acute infection, and this correlated with complement activation, which is consistent with previous reports [43, 44]. Therefore, early interventions that target the complement immune response during acute infection in severe vaccination‐COVID‐19 are of utmost importance to prevent thrombosis and excessive inflammatory responses that are caused by complement hyperactivation. However, given the limited size our cohort, the impact of sex and age on complement activation could not be studied. Additionally, considering the retrospective nature of the study, data on complement intervention were lacking, and future large‐scale cohort studies with intervention designs are necessary.

## Author Contributions

Zhongfang Wang, Nanshan Zhong, Yunhui Zhang, and Zhuxiang Zhao conceived this study. Zhongfang Wang and Jinpeng Cao interpret data and drafted the manuscript. Jinpeng Cao, Gang Yang, Deyi Huang, and Shiqin Jin performed all the experiments. Tingting Cui, Xiaoyun Yang, Mingzhu Huang, Xiaoling Su, Siyi Liu, Yingjiao Xia, Shidong Deng, Deyi Huang, Shiqin Jin, and Chengna Luo gathered the clinical specimens and data. Nanshan Zhong, Yunhui Zhang, Zhuxiang Zhao, Jian Qin, and Gang Yang recruited the cohort and carried out clinical treatments. Zhongfang Wang, Nanshan Zhong, Yunhui Zhang, and Zhuxiang Zhao made critical revision of the manuscript. All authors read and approved the final manuscript.

## Conflicts of Interest

The authors declare no conflicts of interest.

## Supporting information

SUPPLEMENTAL_FIGURE_LEGENDS‐20260106

jmv70863‐sup‐0002.

Tables_S1‐S4‐20251225.

## Data Availability

The data that support the findings of this study are available from the corresponding author upon reasonable request.
