# Supplementary material for: Complement Hyperactivation Is Mediated by Alternative and Lectin Pathways During Early Phase of Severe Vaccination‐Omicron BA.5 Infection
Source: J Med Virol. 2026 Mar 7;98(3):e70863. doi: 10.1002/jmv.70863 (PMC12967031; doi:10.1002/jmv.70863)
Supplement: Supplementary file 1 — SUPPLEMENTAL_FIGURE_LEGENDS‐20260106 [file JMV-98-e70863-s001.docx]

**SUPPLEMENTAL FIGURES AND FIGURE LEGENDS：**

**Manuscript title: Complement hyperactivation is mediated by alternative and lectin pathways during early phase of severe vaccination-Omicron BA.5 infection**

**Authors:**

Jinpeng Cao^1, 2†^; Gang Yang^2, 3†^; Tingting Cui^1, 2, 4†^; Jian Qin^3†^; Deyi Huang^1^; Shiqin Jin^1^; Xiaoyun Yang^1, 2^; Mingzhu Huang^1^; Xiaoling Su^1, 2^; Siyi Liu^1^; Yingjiao Xia^3^; Shidong Deng^1^; Chengna Luo^5^; Zhuxiang Zhao^6*^; Yunhui Zhang^3*^; Nanshan Zhong^1, 2, 3*^; Zhongfang Wang^1, 2*^

**Affiliations:**

^1^State Key Laboratory of Respiratory Disease & National Clinical Research Center for Respiratory Disease, Guangzhou Institute of Respiratory Health, the First Affiliated Hospital of Guangzhou Medical University, Guangzhou Medical University, Guangzhou, China; ^2^Guangzhou National Laboratory, Guangzhou International Bio Island, Guangzhou, China; ^3^The Affiliated Hospital of Kunming University of Science and Technology. Department of Pulmonary and Critical Care Medicine, The First People's Hospital of Yunnan Province, Kunming, Yunnan, China; ^4^Department of Clinical Laboratory, State Key Laboratory of Respiratory Disease and National Clinical Research Centre for Respiratory Disease, The First Affiliated Hospital of Guangzhou Medical University, Guangzhou Medical University, Guangzhou, Guangdong, China. ^5^South China University of Technology, Guangzhou, China. ^6^Department of Infectious Disease, Respiratory and Critical Care Medicine, Guangzhou First People's Hospital, Guangzhou Medical University, Guangzhou, Guangdong, China.

^†^These authors contributed equally to this work.

***Corresponding authors:** zhaozhuxiang@126.com

[yunhuizhang3188@126.com](mailto:yunhuizhang3188@126.com)

nanshan@vip.163.com

[wangzhongfang@gird.cn](mailto:wangzhongfang@gird.cn) (lead contact)

**Supplementary Figure Legends：**

**Figure S1 Supplementary data supporting the main findings. (A)** Study design and cohort characteristics. **(B)** Comparison of sC5b-9 levels in unvaccinated and uninfected healthy donors and healthy donors who received three doses of inactivated vaccines approximately 4 and 8 months ago. The magenta number indicates the geometric mean titers (GMT) of complement factor sC5b-9 in healthy donors. The red dotted line represents the median and interquartile range (IQR). Each black dot represents one donor, HC No Vaccination (n=54), I-I-I 4 Months (n=19) and I-I-I 8 Months (n=17). Comparisons were performed using the Mann–Whitney *U* test and showed no significant intergroup difference. **(C)** Spearman correlation analysis of complement factor Bb and Factor B in vaccinated patients with different COVID-19 severities during acute infection. **(D-E)** Spearman correlation analysis of complement factor MBL/C1q and C-reactive protein in vaccinated patients with different COVID-19 severities during acute infection.
