# Supplementary figures and images for: Complement Hyperactivation Is Mediated by Alternative and Lectin Pathways During Early Phase of Severe Vaccination‐Omicron BA.5 Infection
Source: J Med Virol. 2026 Mar 7;98(3):e70863. doi: 10.1002/jmv.70863 (PMC12967031; doi:10.1002/jmv.70863)

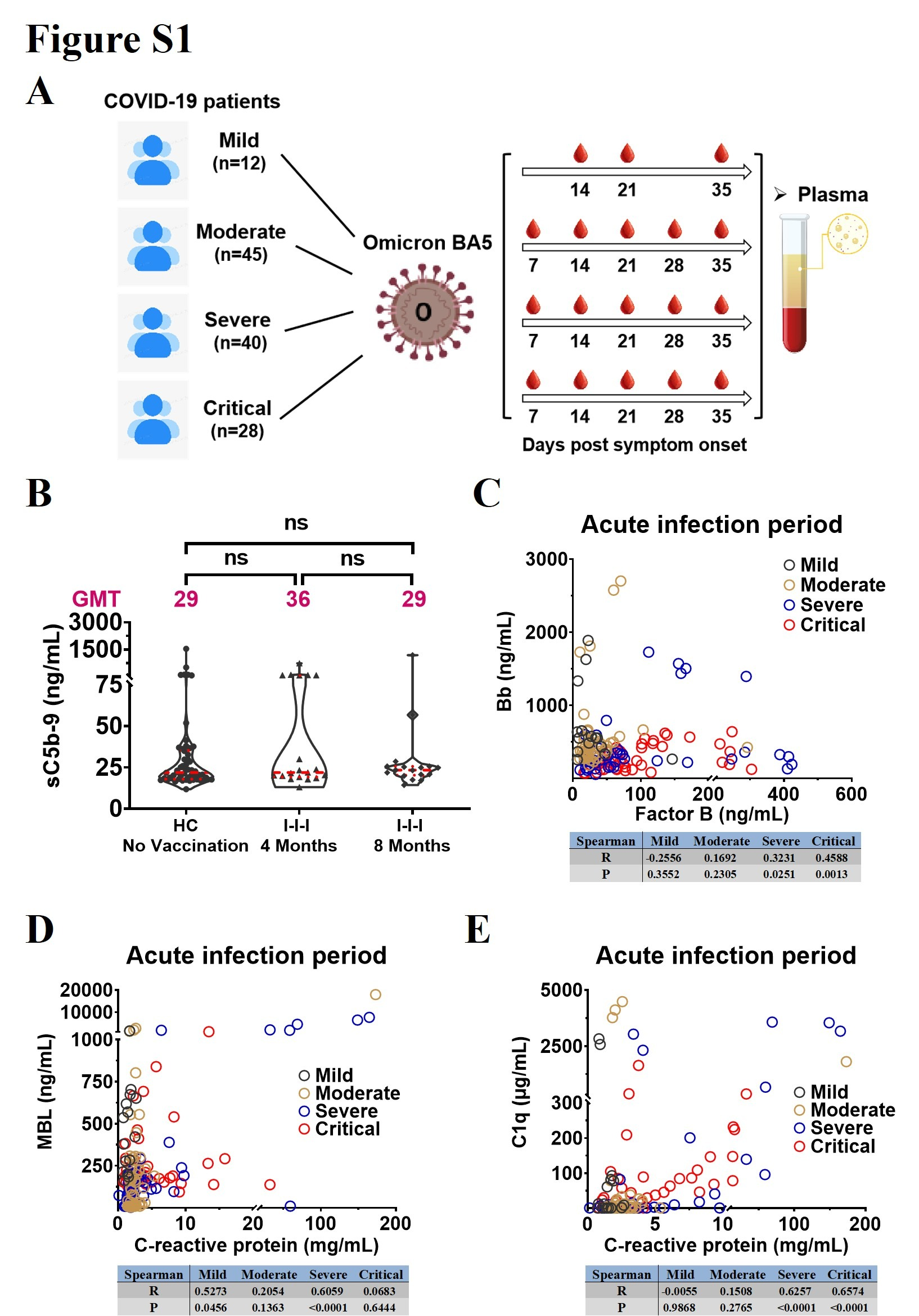

Supplement: Supplementary file 2 — jmv70863‐sup‐0002. [file JMV-98-e70863-s003.png]
